# Comment on the association of preoperative frailty with the risk of postoperative delirium in older patients undergoing hip fracture surgery: a prospective cohort study

**DOI:** 10.1007/s40520-024-02760-4

**Published:** 2024-05-13

**Authors:** Xiao-Ming Zhang, Ya-Nan Gu

**Affiliations:** Department of emergency, the people’s hospital of baoan Shenzhen, Shenzhen, China

Dear editor

We read with great interest the recent study investigating the relationship between frailty and postoperative delirium (POD) in older individuals undergoing hip fractures [1]. The authors conducted an excellent study and found that older adults with frailty had a 5.16-fold higher risk of POD compared to those without frailty. Additionally, the authors found that age was also a risk factor for POD in older people. This study stresses the importance of screening for frailty before surgical procedures and taking measures to reduce frailty in order to mitigate the risk of POD in older people with hip fracture surgery. We applaud the authors for exploring this important association. However, we believe that certain issues require discussion.

Firstly, in the methods section, we noticed that the authors used a cumulative deficit model to calculate the Frailty Index (FI) and classified people as frail or non-frail, with a cut-off value of 0.25. It is crucial to specify which items were included in calculating the Frailty Index. A previous study used a 26-item Frailty Index, consisting of various chronic diseases, activities of daily living, malnutrition, hemoglobin serum levels, and polypharmacy, to confirm frailty [2]. The results found that frailty was a risk factor for POD, with the unadjusted OR being 7.61 (95%CI: 5.66–10.25). We suppose that the various variables for the Frailty Index might influence the association between frailty and delirium. Therefore, it would be very helpful for the reader to fully understand the findings if the authors could provide detailed information on the total of 40 deficit variables in Feng’s study [1].

Secondly, when the authors aimed to identify the independent association between frailty and POD, it was crucial to adjust for potential confounders. We believe that baseline cognitive function was an important confounder that should be assessed. A previously published study that explored the association between frailty and POD in older orthopedic trauma patients found that baseline cognitive function was associated with both frailty and POD [3]. Another study exploring the same association for the same population with hip fractures also found that cognitive function was associated with frailty and POD [2]. Therefore, cognitive function should be considered a potential confounder that needs to be adjusted.

In addition, adopting other important characteristics and clinical variables, such as type of anesthesia, type of fractures, surgical delay ( > = 48 h), number of daily drugs, and mobility status is encouraged. These variables have been reported to associate with frailty or POD. Only by fully adjusting for these potential confounders can the true independent association between frailty and POD among older people with hip fracture surgery be confirmed.

Furthermore, we suggest that the authors conduct a univariate analysis for POD. The results of the univariate analysis of POD can assist readers in identifying which risk factors influence POD, and these results also assist the authors in confirming which variables should be included in the multivariate logistic regression.

We notice that the authors used albumin < 34 g/L as an indicator of malnutrition. In fact, using albumin as an indicator of malnutrition is controversial. A previous study found that hepatic proteins were more likely indicators of morbidity and mortality rather than nutritional status [4]. Therefore, if the authors could use other assessment tools such as NRS 2002, the Malnutrition Universal Screening Tool, or the Short-Form Mini Nutritional Assessment to confirm malnutrition, it would more accurately reflect the nutritional status of older people with hip fractures.

In conclusion, the authors have conducted an excellent study among Chinese older individuals with hip fracture surgery, providing valuable insights for medical staff to early screen for frailty among this special population. Additionally, this study can remind medical staff to consider frailty assessment as an important preoperative evaluation for elderly patients with hip fractures, potentially reducing the incidence of POD. We hope our suggestions will assist the authors in enhancing the quality of this important study.

## Data Availability

No datasets were generated or analysed during the current study.
